# Supplementary material for: Identification of photoperiod-induced specific miRNAs in the adrenal glands of Sunite sheep (Ovis aries)
Source: Front Vet Sci. 2022 Jul 22;9:888207. doi: 10.3389/fvets.2022.888207 (PMC9354845; doi:10.3389/fvets.2022.888207)
Supplement: Supplementary file 1 [file Data_Sheet_1.ZIP › Supplementary file/miRDeep2 file.zip!/miRDeep2 file/Know_Structure/SPLP42A1/oar-let-7f.pdf]

miRBase precursor : oar-let-7f
Total read count : 659189
oar-let-7f read count : 659076
remaining reads : 113

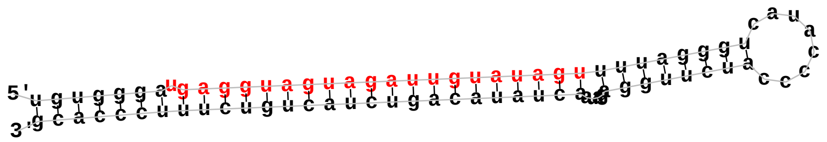

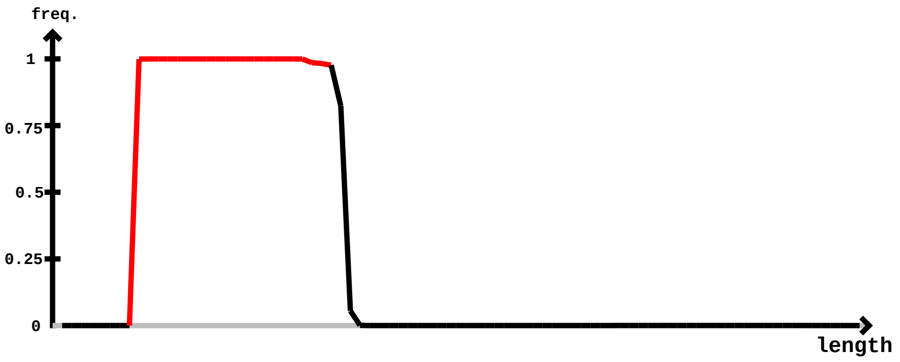

oar-let-7f

```
5'- ugugggaugagguaguagauuguauaguuuuagggucauaccccaucuuggagauaacuauacagucuacugucuuucccacg   -3'    exp
    .((((((.((((((((((((((((((((((((((((.........))))))))....)))))))))))))))))))))))))).   reads     mm        sample
    ......Gugagguaguagauuguauagu........................................................   2         1         seq
    ......augagguaguagauuguauagu........................................................   9         0         seq
    ......augagguaguagauuguauaguu.......................................................   26        0         seq
    ......Gugagguaguagauuguauaguu.......................................................   7         1         seq
    .......ugagAuaguagauugua............................................................   3         1         seq
    .......ugagguaguagauuguU............................................................   3         1         seq
    .......ugagguaguagauugua............................................................   235       0         seq
    .......ugaAguaguagauugua............................................................   1         1         seq
    .......ugagguaguagauugAau...........................................................   2         1         seq
    .......ugagguaguagauuguUu...........................................................   6         1         seq
    .......ugagguaguagauuguau...........................................................   9027      0         seq
    .......ugaAguaguagauuguau...........................................................   73        1         seq
    .......ugagguaguUgauuguau...........................................................   8         1         seq
    .......ugagguaguagauuguaua..........................................................   1950      0         seq
    .......ugagguaguagauuguauag.........................................................   4132      0         seq
    .......ugagguaguagauugAauagu........................................................   23        1         seq
    .......ugagguaguagauuguauagu........................................................   100519    0         seq
    .......ugagguaguagauuguauaguu.......................................................   506255    0         seq
    .......ugagguaguagauuguauaguuG......................................................   2108      1         seq
    .......ugagguaguagauuguauaguuu......................................................   33870     0         seq
    .......ugagguaguagauuguauaguuGu.....................................................   90        1         seq
    .......ugagguaguagauuguauaguuuu.....................................................   304       0         seq
    .......ugagguaguagauuguauaguuuua....................................................   3         0         seq
    ........gagguaguagauuguau...........................................................   2         0         seq
    ........gagguaguagauuguaua..........................................................   1         0         seq
    ........gagguaguagauuguauag.........................................................   2         0         seq
    ........gagguaguagauuguauagu........................................................   44        0         seq
    ........gagguaguagauuguauaguu.......................................................   270       0         seq
    ........gagguaguagauuguauaguuu......................................................   19        0         seq
    ........gagguaguagauuguauaguuG......................................................   4         1         seq
    .........agguaguagauuguauaguu.......................................................   6         0         seq
    .........agguaguagauuguauaguuG......................................................   9         1         seq
    .........agguaguagauuguauaguuGu.....................................................   55        1         seq
    ..........gguaguagauuguauaguu.......................................................   3         0         seq
    ...........guaguagauuguauaguu.......................................................   4         0         seq
```

oar-let-7f

```
uguggga ugagguaguagauuguauagu uuuagggucauaccccaucuuggagauaacuauacagucuacugucuuucccacg
............uaguagauuguauaguC......................................................    1    1    seq
............................uuuagggucauaccccaucuuggaga.............................    1    0    seq
.............................uuagggucauaccccaucuuggag..............................    1    0    seq
......................................................uaacuauacagucuacugucuuu......    1    0    seq
.........................................................cuauacagucuacugucuu.......    1    0    seq
.........................................................cuauacagucuacugucuuu......    13   0    seq
.........................................................cuauacagucuacugucuuuc.....    26   0    seq
.........................................................cuauacagucuacugucuuucc....    70   0    seq
```
